# TWIST1 and TWIST2 promoter methylation and protein expression in tumor stroma influence the epithelial-mesenchymal transition-like tumor budding phenotype in colorectal cancer

**DOI:** 10.18632/oncotarget.2716

**Published:** 2014-11-26

**Authors:** José A. Galván, Melina Helbling, Viktor H. Koelzer, Mario P. Tschan, Martin D. Berger, Marion Hädrich, Beat Schnüriger, Eva Karamitopoulou, Heather Dawson, Daniel Inderbitzin, Alessandro Lugli, Inti Zlobec

**Affiliations:** ^1^ Translational Research Unit (TRU), Institute of Pathology, University of Bern, Bern 3010, Switzerland; ^2^ Clinical Pathology Division, Institute of Pathology, University of Bern, Switzerland; ^3^ Experimental Pathology Division, Institute of Pathology, University of Bern, Switzerland; ^4^ Department of Medical Oncology, Bern University Hospital, Bern, Switzerland; ^5^ Departments of Visceral Surgery and Medicine, Bern University Hospital, Bern, Switzerland; ^6^ Department of Surgery, Tiefenau Hospital, Bern, Switzerland

**Keywords:** tumor microenvironment, methylation, budding, pyrosequencing, Twist

## Abstract

Tumor budding in colorectal cancer is likened to an epithelial-mesenchymal transition (EMT) characterized predominantly by loss of E-cadherin and up-regulation of E-cadherin repressors like TWIST1 and TWIST2. Here we investigate a possible epigenetic link between TWIST proteins and the tumor budding phenotype. TWIST1 and TWIST2 promoter methylation and protein expression were investigated in six cell lines and further correlated with tumor budding in patient cohort 1 (*n* = 185). Patient cohort 2 (*n* = 112) was used to assess prognostic effects. Laser capture microdissection (LCM) of tumor epithelium and stroma from low- and high-grade budding cancers was performed. In colorectal cancers, TWIST1 and TWIST2 expression was essentially restricted to stromal cells. LCM results of a high-grade budding case show positive TWIST1 and TWIST2 stroma and no methylation, while the low-grade budding case was characterized by negative stroma and strong hypermethylation. TWIST1 stromal cell staining was associated with adverse features like more advanced pT (*p* = 0.0044), lymph node metastasis (*p* = 0.0301), lymphatic vessel invasion (*p* = 0.0373), perineural invasion (*p* = 0.0109) and worse overall survival time (*p* = 0.0226). Stromal cells may influence tumor budding in colorectal cancers through expression of TWIST1. Hypermethylation of the tumor stroma may represent an alternative mechanism for regulation of TWIST1.

## INTRODUCTION

Epithelial-mesenchymal transition (EMT) in cancer refers to a reversible and dynamic process by which cells tend to lose epithelial characteristics in favor of a more mesenchymal phenotype [1]. This includes on the one-hand de-differentiation and loss of cell adhesion, while on the other resistance to apoptosis and a gain in migration and invasion potential.

Central to this process is E-cadherin, a cell-cell adhesion molecule that provides an anchor between the basolateral membrane of adherens junctions and the actin cytoskeleton [2]. Loss of E-cadherin is considered a pre-requisite for EMT favoring tumor cell dissemination and metastasis [3]. Although mutation of the E-cadherin gene (CDH1) and CpG island hypermethylation of the CDH1 promoter have been described, E-cadherin may also be inhibited through transcriptional repression by other factors such as ZEB family members and the TWIST-family of bHLH proteins [4–10]. TWIST itself may be regulated through gene promoter hypermethylation leading to absence of the protein in HCT116 cells [11]. Moreover, epigenetic regulation also impacts microRNAs that regulate gene networks involved in EMT [12].

In colorectal cancers, loss of membranous E-cadherin is frequently observed along the tumor invasion front, most notably in tumor budding cells [13]. Tumor buds are defined as single tumor cells or small cell clusters (<5) detached from the main tumor body [14]. In addition to nuclear β-catenin and loss of E-cadherin, tumor buds are thought to represent a non-proliferating, non-apoptotic subgroup of cancer cells derived from the process of EMT and highly aggressive in nature as underlined by their migratory and invasive immunohistochemical protein profile [15]. As a prognostic marker, the presence of high-grade tumor budding has shown consistent associations with negative clinicopathological parameters; it is a predictor of lymph node metastasis, distant metastatic disease and is a distinctly unfavorable prognostic factor in patients with colorectal cancer, independently of TNM stage or treatment [14, 16–20]. Furthermore, high-level DNA microsatellite instability (MSI-H) has been associated with more favorable prognosis and little tumor budding in colorectal cancer patients [13] as well as with decreased EMT marker expression in MSI cell lines compared to microsatellite stable (MSS) cells [21].

Although the transcriptional regulation of EMT is well-described and WNT-related protein expression changes in tumor budding cells are known, only sparse information can be found on the possible role of CpG promoter methylation on the promotion/suppression of the EMT-like phenotype. Since the regulation of E-cadherin is so central to the tumor budding process, and given the hypothesis of promoter hypermethylation as a possible regulatory mechanism for TWIST, we investigate the relationship between tumor budding and the CpG methylation status of repressors of E-cadherin, TWIST1 and TWIST2.

## RESULTS

### TWIST1 and TWIST2 methylation patterns in colorectal cancer cell lines

**Figure 1:**
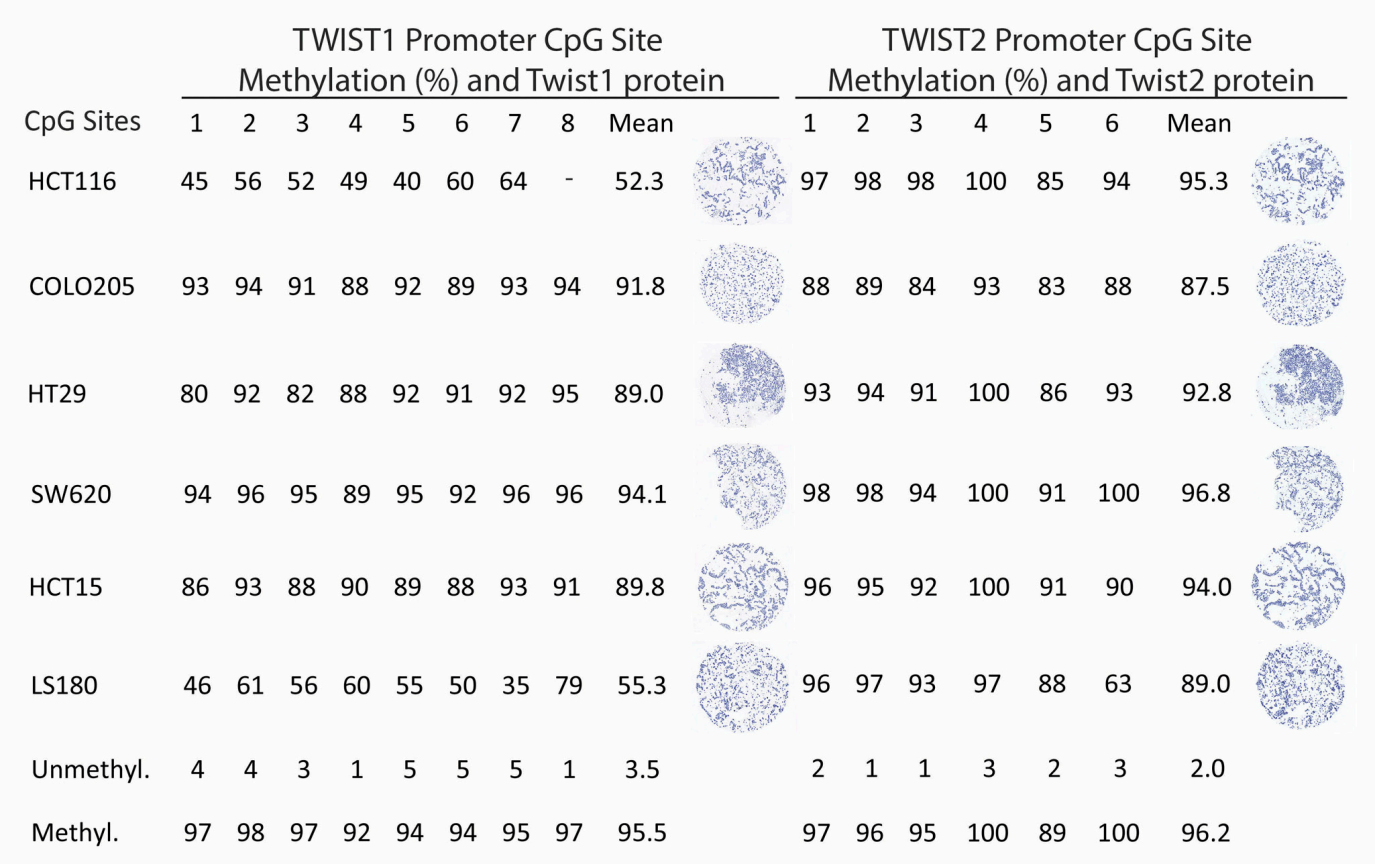
Pyrosequencing and immunohistochemistry data for TWIST1 and TWIST2 in cell lines showing marked hypermethylation across all cell lines and complete negative protein expression (200x magnification, scale bar = 100 μm)

We evaluated TWIST1 and TWIST2 methylation status in six well-established colorectal cancer cell lines and their corresponding immunohistochemistry detection. In all cases, cell lines exhibited significant hypermethylation (range TWIST1: 52.3%–94.1% and TWIST2: 87.5%–96.8%). Immunohistochemistry staining for TWIST1 and TWIST2 were completely negative in all cell lines (Figure 1).

### Correlation of TWIST 1 and TWIST2 with a tumor budding phenotype

**Figure 2:**
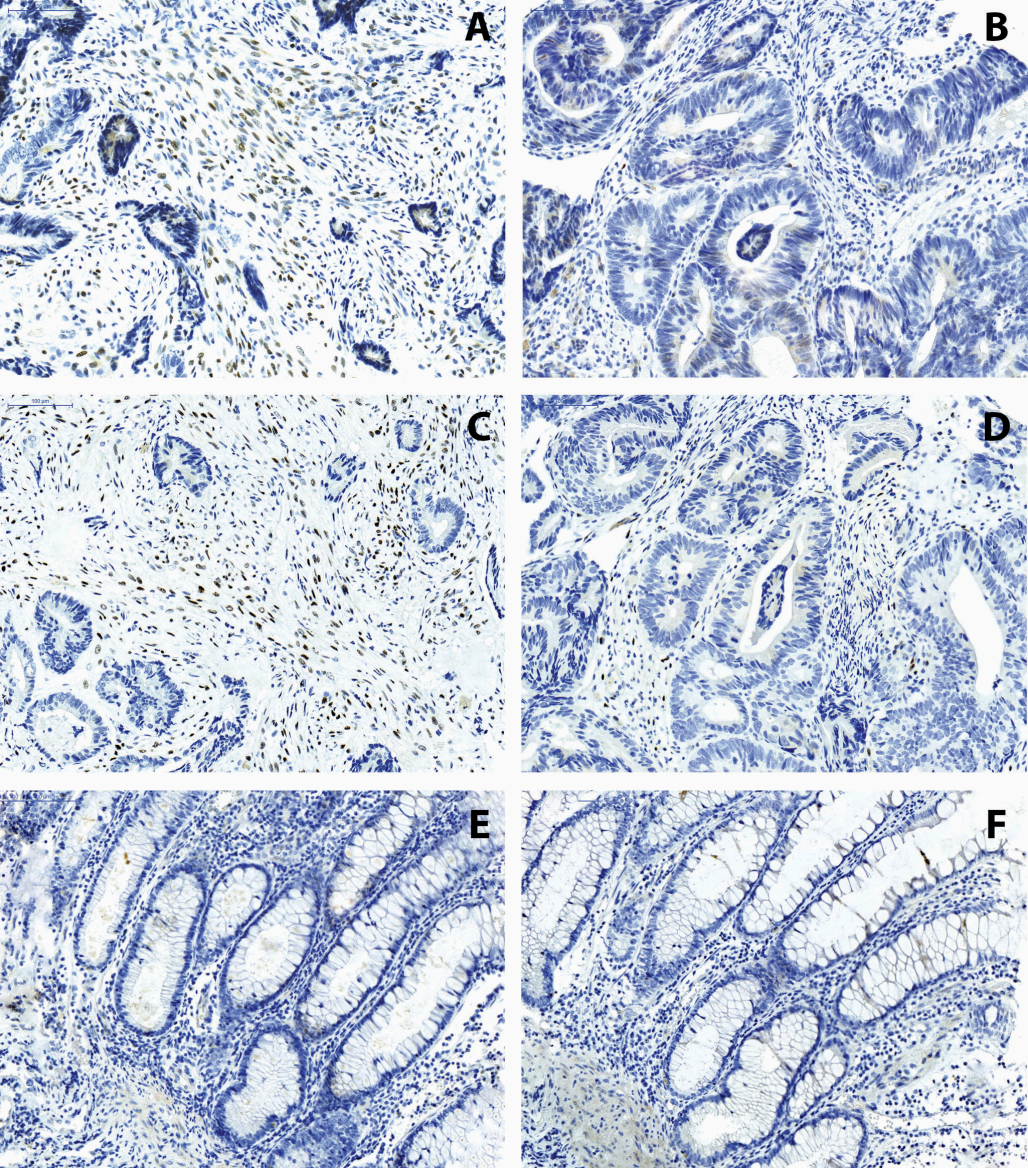
Immunohistochemistry of TWIST1 **(A, B)** and TWIST2 **(C, D)** on colorectal cancers and **(E, F)** normal colonic tissues Expression is found almost exclusively in the stroma and ranges from minimal to extensive (200x magnification, scale bar = 100 μm).

Of the 215 patients included into cohort 1, methylation analysis for TWIST1 was successful in 107 cases and for TWIST2 in 185 cases. TWIST1 and TWIST2 CpG methylation was correlated to protein expression and tumor budding status. Expression was analyzed by tissue microarray in tumor epithelia and tumor stroma, separately. Prominent stromal cell staining for TWIST1 and TWIST2 could be noted with only very infrequent expression within tumor epithelia (Figure 2).

An inverse relationship between methylation of TWIST1 and TWIST2 and high-grade budding was observed. Of note, all 17 cases considered hypermethylated for TWIST1 defined as >75th-percentile (>65% methylation) were found to have no or low-grade tumor budding (Supplementary Table S1). An inverse relationship between higher budding counts and TWIST2 methylation was also noted (*p* = 0.0724) but did not reach statistical significance.

In order to better understand the relationship between tumor budding, methylation and protein expression of TWIST1 and TWIST2, tumor budding status was divided into low (≤10 buds/10HPFs on average) and high (>10 buds/10 HPFs on average) [14]. In high-grade budding cancers, significant inverse correlations between TWIST1 methylation and TWIST1 stromal expression was observed (high-grade *r* = –0.4; *p* < 0.001). Only a weak inverse relationship between TWIST2 methylation and protein expression was found (ranging from –0.08 to –0.11). TWIST1 and TWIST2 expression in stroma were significantly positively correlated in low-grade (*r* = 0.41) and to a lesser extent in high-grade (*r* = 0.26) budding cancers.

### Laser capture microdissection of low-grade and high-grade budding cancers

**Figure 3:**
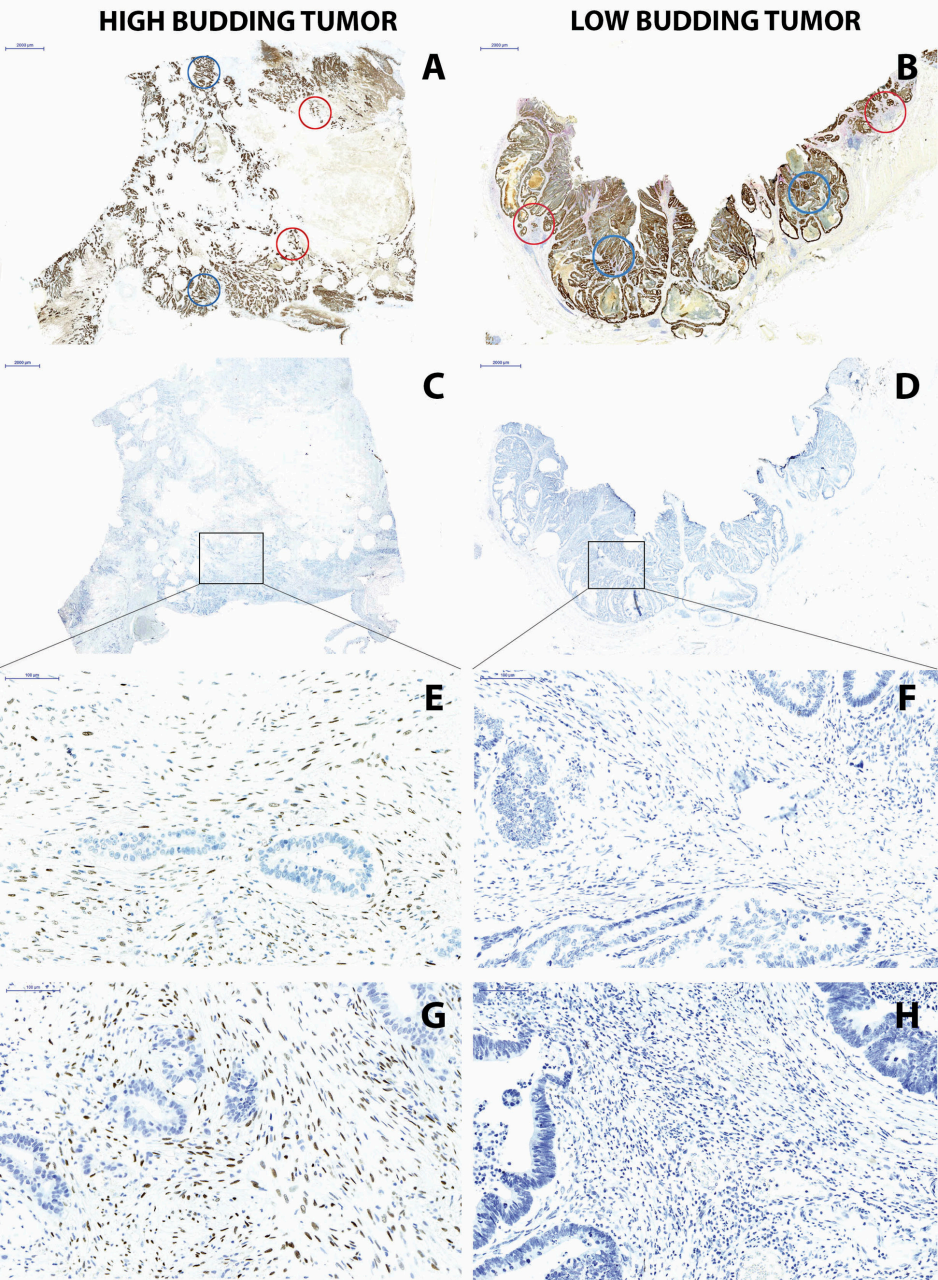
(Left) High-grade budding and (Right) low-grade budding cancer **(A, B)** Pan-cytokeratin staining with designated areas subsequently taken for laser capture microdissection including 2 center (blue) and 2 invasion front (red) and **(C, D)** TWIST1 staining showing designated area for subsequent laser capture microdissection of stroma (10x magnification, scale bar = 2000 μm), showing **(E)** positive TWIST1 staining in high-grade budding cancer and **(F)** negative TWIST1 staining in low-grade budding cancer, **(G)** TWIST2 staining in high-grade budding cancer and **(H)** TWIST2 staining in low-grade budding cancer (200x magnification, scale bar = 100 μm).

We selected 1 case with no tumor budding and 1 case with extensive tumor budding for further analysis. TWIST1 and TWIST2 immunohistochemistry were performed. In both cases, the tumor epithelium was nearly negative with very rare positive cells. In contrast, the high-grade budding tumor showed considerable stromal cell staining of TWIST1 and TWIST2 while the low-grade budding cancers had a mostly negative stroma (Figure 3).

We performed laser capture microdissection of stroma in both cases. In accordance with the immunohistochemistry, we found hypomethylation (3.9% and 6.7%, respectively) of TWIST1 and TWIST2 in the high-grade budding cancer that stained positive for the proteins and a considerable hypermethylation (30.3% and 42.2%, respectively) of TWIST1 and TWIST2 in the low-grade budding tumor that stained negative for the proteins (Table 1).

**Table 1:** Laser capture microdissection results for methylation of TWIST1 and TWIST2 in tumor center, front and stroma in a low-grade and high-grade budding cancer

		TWIST1		TWIST2	
		Protein expression	Mean methylation %	Protein expression	Mean methylation %
**High-grade budding**	Center 1	Negative	1.3	Negative	12.8
	Center 2	Negative	2.9	Negative	32.8
	Front 1	Negative	1.8	Negative	1.3
	Front 2	Negative	1.3	Negative	22.8
	Stroma	**Positive**	**3.9**	**Positive**	**6.7**
**Low-grade budding**	Center 1	Negative	84.1	Negative	88.7
	Center 2	Negative	22.3	Negative	84.5
	Front 1	Negative	97.6	Negative	70.8
	Front 2	Negative	1.0	Negative	98.2
	Stroma	**Negative**	**30.3**	**Negative**	**42.2**

We additionally captured 4 different regions of tumor epithelium per case (2 tumor center and 2 tumor front). Pyrograms can be found in Supplementary Figures S1 and S2. In accordance with our results of a possible inverse relationship between methylation and tumor budding phenotype, we observe a significant hypermethylation of TWIST1 and TWIST2 in correlation with low-grade budding and a hypomethylation of TWIST1 and TWIST2 in the high-grade budding tumor.

### Immunohistochemistry for EMT markers in low-grade and high-grade budding cases

Whole tissue sections from the same low-grade and high-grade budding cancers were immunostained for β-catenin, E-cadherin, CDX2 [22], ZEB1 and ZEB2 and found to exhibit the expected protein phenotype. The high-grade budding cancer showed a nuclear translocation of β-catenin toward the invasion front, loss of membranous E-cadherin, absence of CDX2, and increased ZEB1 and ZEB2 in stromal cells at the invasion front. The low-grade budding cancer, having no buds to analyze, showed the membranous β-catenin, intact E-cadherin, nuclear CDX2 and absence of ZEB2 (Supplementary Figure S3). ZEB1 positive stroma could still be observed in this case.

### Prognostic significance of TWIST1 and TWIST2 in colorectal cancer patients

**Figure 4:**
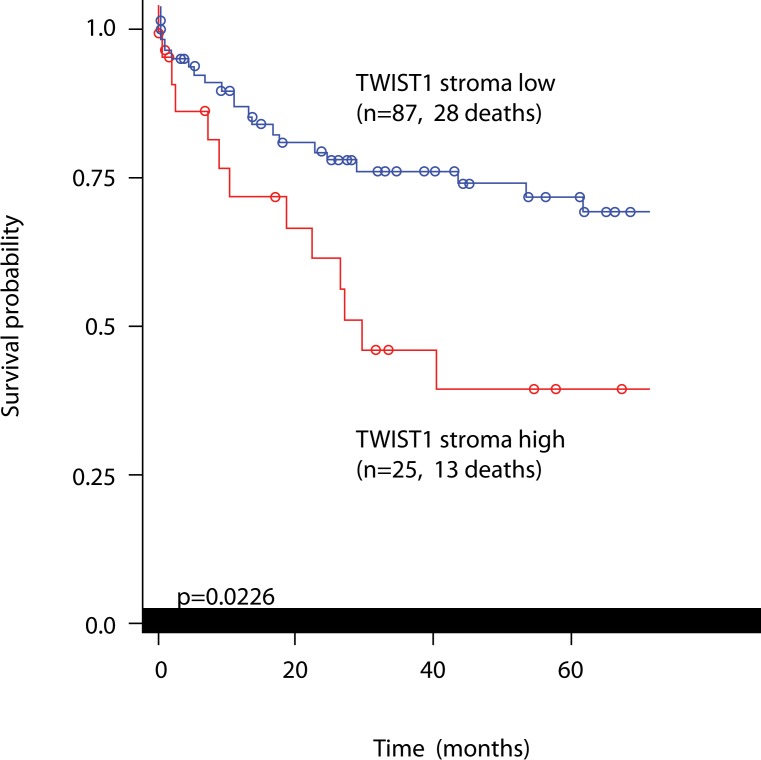
Kaplan-Meier curve showing prognostic differences in patients with low or high TWIST1 stromal cell expression Log-rank test.

Of the 139 patients originally considered in Cohort 2, those receiving a preoperative therapy were excluded from the analysis, leaving 112 patients. TWIST1 and TWIST2 immunohistochemistry was performed on a preoperative biopsy tissue microarray of these patients with full clinicopathological and overall survival time data. Stromal cell staining ranged from minimal to extensive, while only rare TWIST1 expression could be found in tumor cells. Results are found in Table 2. A significant adverse effect of TWIST1 expression was found and related to higher tumor grade (*p* = 0.0229), more advanced pT classification (*p* = 0.0044), lymph node metastasis (*p* = 0.0301), lymphatic vessel invasion (*p* = 0.0373), frequent perineural invasion (*p* = 0.0109) and significantly worse overall time (*p* = 0.0226; Figure 4). This prognostic difference was maintained in multivariable analysis (Table 3). Although a positive correlation between TWIST1 and TWIST2 protein expression was found (*r* = 0.64, *p* < 0.0001), TWIST2 expression was not linked to any prognostic features.

**Table 2:** TWIST1 and TWIST2 stromal cell expression and clinicopathological features; median values used to define low/high, namely 30% TWIST1 and 50% TWIST2

Feature		TWIST1 (*n* = 113)		*P*-value	TWIST2 (*n* = 115)		*P*-value
		Low (*n* = 65; 57.5%)	High (*n* = 48; 42.5%)		Low *n* = 73; 63.5%)	High (*n* = 42; 36.5%)	
Gender	Male	36 (55.4)	30 (63.8)	0.37	39 (53.4)	30 (73.2)	**0.0468**
	Female	29 (44.6)	17 (36.2)		34 (46.6)	11 (26.8)	
Histology	Non-mucinous	57 (87.7)	38 (79.2)	0.1049	64 (86.5)	33 (80.5)	0.3197
	Mucinous	8 (12.3)	10 (20.8)		10 (13.5)	8 (19.5)	
Tumor grade	G1-2	50 (79.4)	26 (59.1)	0.0229	50 (71.4)	26 (66.7)	0.604
	G3	13 (20.6)	18 (40.9)		20 (28.6)	13 (33.3)	
Tumor location	Left	18 (27.7)	20 (43.5)	0.2249	16 (21.9)	22 (55.0)	**0.0016**
	Rectum	16 (24.6)	9 (19.6)		20 (27.4)	5 (12.5)	
	Right	31 (47.7)	17 (37.0)		37 (50.7)	13 (32.5)	
pT	pT1-2	24 (36.9)	6 (12.8)	**0.0044**	20 (27.4)	10 (24.4)	0.7264
	pT3-4	41 (63.1)	41 (87.3)		53 (72.6)	31 (75.6)	
pN	pN0	37 (56.9)	17 (36.2)	**0.0301**	40 (54.8)	18 (43.9)	0.2643
	pN1-2	28 (43.1)	30 (63.8)		33 (45.2)	23 (56.1)	
cM	cM0	45 (72.6)	32 (69.6)	0.7319	24 (77.4)	11 (68.7)	0.5184
	cM1	17 (27.4)	14 (30.4)		7 (22.6)	5 (31.3)	
L	L0	19 (35.9)	7 (16.7)	**0.0373**	17 (28.3)	10 (27.0)	0.8891
	L1-2	34 (64.2)	35 (83.3)		43 (71.7)	27 (73.0)	
V	V0	34 (61.8)	20 (46.5)	0.1306	29 (46.8)	23 (60.5)	0.1815
	V1-2	21 (38.2)	23 (53.5)		33 (53.2)	15 (39.5)	
Pn	Pn0	51 (98.1)	35 (83.3)	**0.0109**	53 (91.4)	33 (86.8)	0.4767
	Pn1	1 (1.9)	7 (16.7)		5 (8.6)	5 (13.2)	
MMR status	Proficient	47 (82.5)	40 (90.9)	0.2228	56 (82.4)	33 (91.7)	0.1984
	Deficient	10 (17.5)	4 (9.1)		12 (17.7)	3 (8.3)	
**Overall survival time**	5-year (95%CI)	69.3 (57–79)	38.1 (18–59)	**0.0226**	63 (51–73)	48 (21–71)	0.8724

**Table 3:** Multivariable survival analysis of TWIST1 protein expression in stroma

Feature		HR (95%CI)	*P*-value
Stroma	Low	1.0	**0.0467**
	High	1.99 (1.01–3.92)	
pT	pT1-2	1.0	0.8425
	pT3-4	0.92 (0.4–2.1)	
pN	pN0	1.0	0.8075
	pN1-2	1.09 (0.55–2.2)	
Therapy	None	1.0	0.0589
	Treated	0.4 (0.2–1.04)	

### TWIST1 and TWIST2 methylation patterns in normal colonic tissue and cancer

In order to determine whether the hypermethylation of TWIST1 and TWIST2 as a mechanism for TWIST1 and TWIST2 down-regulation is restricted to tumor, we included analysis of 10 normal colonic tissues. TWIST1 and TWIST2 were significantly more methylated in cancers in comparison to normal tissues (TWIST1 40.9% versus 13%, *p* = 0.0012, TWIST2 41.4% versus 21%, *p* < 0.0001) with a range from 0–100%. Normal epithelium was only rarely immunoreactive for either protein.

## DISCUSSION

In this study, we investigated the possible link between TWIST1 and TWIST2 methylation in tumor epithelium and tumor stroma, the relationship with TWIST1 and TWIST2 protein expression and a possible role for these genes in promoting a tumor budding phenotype. Our results suggest that the pro-tumor effects of TWIST1 and, to some degree, TWIST2 come from the tumor stroma and that this expression may be regulated at least in part by promoter hypermethylation.

A recent work by Laghi and colleagues investigated TWIST1 in cell lines and samples of colorectal cancer patients [11]. They could show that TWIST1-positive cells were predominantly stromal, acquired a fully mesenchymal phenotype and appearance, and remarkably, showed neoplastic aberrations matching those of the main tumor suggesting that an actual EMT had taken place. Our findings are in line with these results. Firstly, we show that TWIST1 and TWIST2 protein expression are found nearly exclusively in the tumor stroma. Selected low- and high-grade budding cases underlined the extensive expression of TWIST1 and TWIST2 in the stromal compartment only in the high-grade budding case with matching expression of classic hallmarks of EMT in tumor buds including nuclear β-catenin, disrupted E-cadherin and over-expression of ZEB1 and ZEB2 in stromal cells.

Several studies to date have found that increased mRNA expression or protein expression of TWIST1 correlates to adverse clinicopathological features and survival time [11, 23–25]. TWIST1 expression has also been quantified in peripheral blood from patients with colorectal cancer and shown to correlate not only with disease progression but with stem cell marker CD133 expression [26]. Strikingly less is known about TWIST2, particularly in colorectal cancer. TWIST2 may function as a tumor suppressor and has been shown to inhibit formation of a microenvironment conducive to tumor growth in a murine osteosarcoma model [27]. In colorectal cancers, recent results from Yu and colleagues suggest that expression of TWIST2 is a valuable adverse prognostic marker [28]. In cervical cancers, in comparison to TWIST1, TWIST2 may better stratify patients into prognostic subgroups and is shown to induce EMT and cell motility in cell lines [29]. Also in ovarian cancer TWIST2 expression is correlated with disease stage [30]. However these studies on patient samples differ from ours in that immunohistochemistry stains are reported within tumor epithelium, rather than tumor stroma. Our findings using a well-characterized cohort of 112 patients and preoperative cancer biopsies also support an association of TWIST1 expression and adverse clinicopathological features. Our result on biopsy material is novel since these specimens do not normally contain the tumor invasion front. Here we again observe extensive TWIST1 and TWIST2 staining in stromal cells within the main tumor body. However, we can confirm associations with more advanced stage of disease and survival time only for TWIST1.

Our findings also support a possible role for promoter hypermethylation in the regulation of TWIST1 and to some degree TWIST2. To support this statement, the following were performed. First, we assessed methylation patterns in 6 well-established colorectal cancer cell lines and determined them all to have extensive hypermethylation and complete absence of protein. Second, we found strong inverse correlations between TWIST1 methylation and stromal expression of TWIST1 protein in our patient samples. Thirdly, we performed laser capture microdissection to dissect out tumor epithelium from tumor stroma in regions previously stained for TWIST1 and TWIST2 protein. The concordance between stromal expression and methylation status could be confirmed. Other groups have reported on TWIST1 and TWIST2 methylation in colorectal cancer. Celesti and colleagues treated HCT116 cells with a DNA methyltransferase inhibitor. Restoration of TWIST1 RNA and protein could be seen, indicating that promoter methylation may at least to some degree be responsible for the absence of TWIST protein. Hypermethylation in cancers in comparison to normal tissues is observed for TWIST1 [25]. Moreover, Ashktorab and colleagues, performing an extensive bioinformatics work on CpG island Methylator Phenotype, identified TWIST1 as markedly hypermethylated in colorectal cancer [31]. Other groups report methylation percentages of >40% across these tumors [32, 33]. Our results confirm these findings not only for TWIST1 but also TWIST2. Methylation as a regulatory mechanism for TWIST1 could be confirmed by Okada et al on cell lines, but not in patient samples [25]. We hypothesize that discrepancies between methylation and expression among patient samples of colorectal cancer may be due to location of TWIST1 stained cells, namely in the tumor stroma, rather than tumor epithelium.

There are many studies that have identified several pathways that control Twist1 expression in tumors, which include NF-κB and STAT3 mediated cytokine signaling [34, 35], induction through hypoxia-inducible factor 1α [36] and the TGFβ pathway [37, 38] as well as post-transcriptional regulatory mechanism by miRNAs [12, 39] but in normal colorectal mucosa these mechanisms are unknown. We observed lack of correlation between hypermethylation and protein expression in normal colorectal mucosa may suggest that hypermethylation as a regulatory mechanism of TWIST1 and TWIST2 may be restricted to the tumor microenvironment. These results are supported by others [25, 33]. Other mechanisms of TWIST1 regulation in normal tissue may include post-translational histone modifications, however this aspect still remains to be elucidated. Nonetheless, in normal colorectal mucosa hypermethylation seems not to be implicated in the Twist1 regulation.

This study is novel for several reasons. 1) To our knowledge, this may be the first study to perform methylation analysis from stromal tissue. We further underline here the feasibility of such an analysis. 2) We perform an extensive evaluation of TWIST1 and TWIST2 hypermethylation and corresponding protein expression on normal colonic tissues, established cell lines, a large number of human colorectal cancer specimens. 3) We show that extensive tumor budding may take place in the context of a TWIST1 (and TWIST2) positive stroma and that such a stroma demonstrates hallmarks of EMT. 4) We perform TWIST1 and TWIST2 evaluations on preoperative biopsy material and are able to underline the prognostic effect of TWIST1, even within the main tumor body.

This study may also have several limitations. Although the correlation between TWIST1 methylation and protein expression is strong, the same is not true for TWIST2. This may be due to the wide range of possible sequences to target during CpG analysis. Secondly, no methylation analysis was performed on Cohort 2. The aim of this second cohort was however to investigate the prognostic effect of the proteins rather than determine correlations with methylation. Since patients with mismatch repair deficient (i.e, MSI-H) colon cancers typically exhibit little tumor budding, one would expect an association between MMR-deficiency and low TWIST expression. Although our results do not reach statistical significance possibly due to the relatively low number of cases in this series, 10/14 MMR-deficient cancers were low for TWIST1 while 12/15 deficient cases were low for TWIST2.

Questions remain as to the timing and mechanism of action at the tumor/stroma interface. Presumably, the most favorable (possibly default) state for the tumor is one in which TWIST1 and TWIST2 are not prevented from being expressed, thus allowing these genes to exert their functions and facilitating and progression. A role for hypermethylation in counteracting the formation of tumor budding would suggest this to be an early event in tumorigenesis, a hypothesis which remains to be determined.

The results of this study suggest that TWIST1 and to a lesser degree TWIST2 expressed within the tumor stroma could contribute to the EMT-like tumor budding phenotype in colorectal cancers. Our evidence suggests a possible role for promoter methylation as a mechanism of TWIST1 regulation.

## METHODS

### Patients

Two retrospective colorectal cancer patient cohorts were entered into this study. Cohort 1 included 215 non-consecutive patients treated at the Fourth Department of Surgery, University of Athens Medical School in Athens, Greece, between 2002 and 2007. Cohort 2 included 139 non-consecutive patients treated at the Insel Hospital, Bern, Switzerland between 2002 and 2011. Detailed information on both cohorts can be found in Supplementary Table S2. Patient gender, age at diagnosis, tumor location, post-operative and pre-operative therapy and **overall** survival time were obtained from patient records. All cases were re-reviewed on H&E stained slides by expert gastro-intestinal pathologists. Features included the pT, pN, pM, tumor grade, histological subtype, venous invasion, perineural invasion (Pn) and lymphatic invasion. For all cases, mismatch repair (MMR) status was determined by evaluating the proteins MLH1, MSH2 and MSH6 (Cohort 1) and additionally PMS2 (Cohort 2). Tumor budding was evaluated using the 10-hotspot method [14]. Briefly, pan-cytokeratin immunohistochemistry (AE1/AE3) was performed on whole tissue sections from each case. The slide was scanned at low-magnification and the 10 densest regions of tumor budding were identified and counted using high-power fields. The average number of buds across these 10 fields was recorded.

### Specimen characteristics

Tumors from both Cohorts 1 and 2 were fixed in 10% buffered formalin and embedded in paraffin. Representative tumor blocks from all cases were retrieved from the corresponding institutes of pathology and used for tissue microarray construction and molecular analysis. In addition, 10 normal colonic tissue blocks were identified in order to obtain baseline comparisons for methylation analysis.

### Tissue microarray construction

Tissue microarray construction was performed using a next-generation Tissue Micro Array (ngTMA) approach [40]. Briefly, an H&E slide from 1 representative tumor block per case was freshly sectioned and scanned using a Pannoramic P250 scanner (3DHistech, Hungary). Next, digital slides were annotated using a TMA tool (Pannoramic Viewer, 3DHistech, Hungary). For cohort 1, the digital slides of each surgical resection were annotated using a 0.6mm tool as follows: 3 annotations of tumor center, 3 of tumor front, and 3 of tumor buds, wherever possible. For cohort 2, preoperative biopsy material from each case was annotated using a 1mm TMA tool. These characteristics are summarized in Supplementary Table S2. Next, annotated regions were punched out using an automated tissue microarray and transferred into corresponding recipient ngTMA blocks (TMA Grandmaster, 3DHistech, Hungary). Ethics committee approval was obtained from the University of Athens (cohort 1) and Insel Hospital (cohort 2, Registration number 07-10-13).

### Immunohistochemistry

Both ngTMAs for Cohorts 1 and 2 were immunostained for TWIST1 and TWIST2 using an automated Leica Bond Rx instrument with the following conditions and antibodies: TWIST1, pre-treatment in citrate buffer at 100°C for 30 minutes, Abcam, rabbit polyclonal, dilution 1:25; TWIST2, pre-treatment with Tris buffer at 95° for 30 minutes, Abcam mouse monoclonal, clone 2C1a, dilution 1:75. Counterstaining was performed with hematoxylin.

Additionally, selected cases underwent immunohistochemistry for markers known to be involved in EMT, namely β-catenin (Abcam, clone E247, pre-treatment in Tris buffer, 95°C for 30 minutes, 1:500), E-cadherin (Dako, clone NCH38, pre-treatment in Tris buffer, 95°C for 30 minutes, 1:200), CDX2 (Novocastra, AMT28, pre-treatment in Tris buffer 95°C for 30 minutes, 1:200), ZEB1 (Sigma-Aldrich, pre-treatment in citrate buffer 100°C for 20 minutes, 1:600) and ZEB2 (Sigma-Aldrich, pre-treatment in Tris buffer 95°C for 60 minutes, 1:100).

### Methylation analysis

From all cases in cohort 1 and 10 cases of healthy tissues performed in parallel, DNA was extracted. For tumors, this was performed by marking the corresponding H&E slides in areas of invasive cancer. A minimum of five whole tissue sections from each case were cut from corresponding paraffin-embedded tissue blocks at 4μm. Slides were scratched using a scalpel in designated regions. DNA was extracted using standard protocols (QIAamp® DNA FFPE Tissue, Qiagen). Bisulfite conversion for subsequent methylation analysis was undertaken (EpiTect® Bisulfite Conversion Kit, Qiagen). PCR was performed using the Pyromark PCR kit (Qiagen) with the following primer sequences for TWIST1 (Entrez Gene ID: 7291) (Microsynth): Forward (biotinylated) 5′-GAAGTTGGAGGGTTGAGG-3′, Reverse 5′- AACTAAACACC TCCTACATCATCT CT-3′ and Sequencing 5′- ACACCTCCTAC ATCATCTCTC-3′. The PCR conditions were as follows: activation step at 95°C for 15 min, denaturation 30 sec at 94°C, annealing 42 cycles of 30 sec at 56°C, and extension 30 seconds at 72°C and final extension 10 min at 72°C. The sequence to analyze was RAACRACRAC RCRTAACCTC RCRAACCCRA AACAAAAAAA AAAAAC and contained 8 CpG sites. The region is located on chromosome 7 at region 19156483-19157779. For TWIST2 (Entrez Gene ID: 117581), a CpG assay from Qiagen was tested (TWIST2_01, sequence to analyze: YGGYGYGTT GATTGGTYGYGGT GGTYGGGGGT) with 6 CpG sites. The region of interest is found within the CpG island located on chromosome 2 at 239 756 376-239 758 300. After PCR, fragment analysis was carried out using a Qiaxcel system (Qiagen). Pyrosequencing was performed using a PyroMark Q24. Bisulfite controls were included into the pyrogram for each assay manually to ensure complete conversion of DNA. In addition, a control oligo and water control was used in every pyrosequencing run as well as appropriate methylated and unmethylated controls (Epitec® PCR Control DNA; Qiagen).

### Colorectal cancer cell lines

In addition to colorectal cancer tissues, six well-established human colon cancer cell lines were included in this study (HCT15, SW620, LS180, HCT116, COLO205, and HT29). Cells were grown in DMEM (HT29), RPMI-1640 (HCT15, SW620, and Colo205), McCoy's (HCT116), or EMEM (LS180) cell culture medium (Sigma-Aldrich, Buchs, Switzerland) supplemented with 10% fetal bovine serum, L-Glutamine and Non-Essential Amino Acids (Sigma-Aldrich) at 37°C in a 5% CO_2_ humified atmosphere. No Antibiotics were used. Genomic DNA was extracted (DNeasy Blood & Tissue Kit, Qiagen) and pyrosequencing for TWIST1 and TWIST2 was undertaken as mentioned above. Cell line pellets were fixed, paraffin-embedded and immunohistochemistry for TWIST1 and TWIST2 was carried out.

### Laser capture microdissection of low and high-grade budding case

In order to analyze areas of tumor stroma and tumor epithelium separately and precisely, 2 colorectal cancers were selected from cohort 2 known to represent a no/low-grade tumor budding case and a high-grade budding case after pan-cytokeratin staining of whole tissue sections. Next, these cases underwent immunohistochemistry for TWIST1 and TWIST2 followed by laser capture microdissection (Zeiss). Briefly, 5×5um sections were cut and placed onto irradiated PEN-membrane slides (Zeiss). Laser capture was performed using a PALMRobo V4.2. DNA extraction (QIAmp DNA Micro Kit, Qiagen), bisulfite conversion, PCR and pyrosequencing for TWIST1 and TWIST2 was performed on 2 areas of tumor center, 2 areas of invasion front and 1 large area of tumor stroma for each case.

### Statistical analysis

Descriptive statistics using means, median and quartiles were performed. Correlation between ranked variables was analyzed using Spearman's correlation coefficient (r). Chi-Square or Fisher's Exact tests were used to analyze categorical variables. The non-parametric Wilcoxon Rank Sum or Kruskal-Wallis tests were used for continuous variables. Kaplan-Meier curves and log-rank tests were applied to detect overall survival time differences. After verifying the proportional hazards assumption, Cox regression analysis for multivariable survival time analysis was performed. *P*-values <0.05 were considered statistically significant. All analyses were carried out using SAS V9.2 (the SAS Institute, Cary, NC).

## SUPPLEMENTARY FIGURES AND TABLE


