# Supplementary material for: TWIST1 and TWIST2 promoter methylation and protein expression in tumor stroma influence the epithelial-mesenchymal transition-like tumor budding phenotype in colorectal cancer
Source: Oncotarget. 2014 Nov 26;6(2):874–85. doi: 10.18632/oncotarget.2716 (PMC4359262; doi:10.18632/oncotarget.2716)
Supplement: Supplementary file 1 [file oncotarget-06-874-s001.pdf]

SUPPLEMENTARY FIGURES AND TABLES

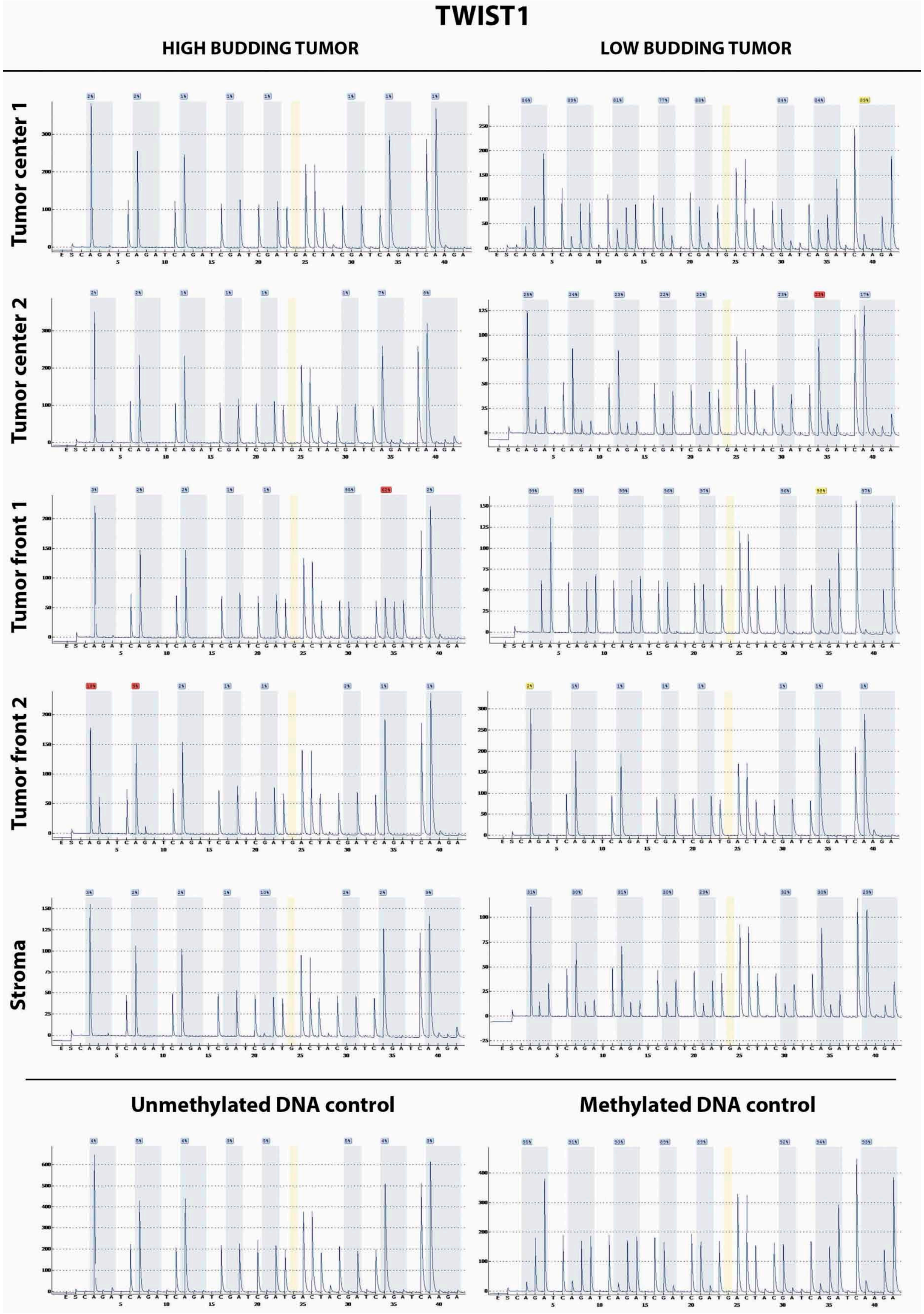

Supplementary Figure S1: Pyrograms showing successful TWIST1 methylation analysis of laser captured tumor center, tumor invasion front and tumor stroma in a low-grade and high-grade budding cancer

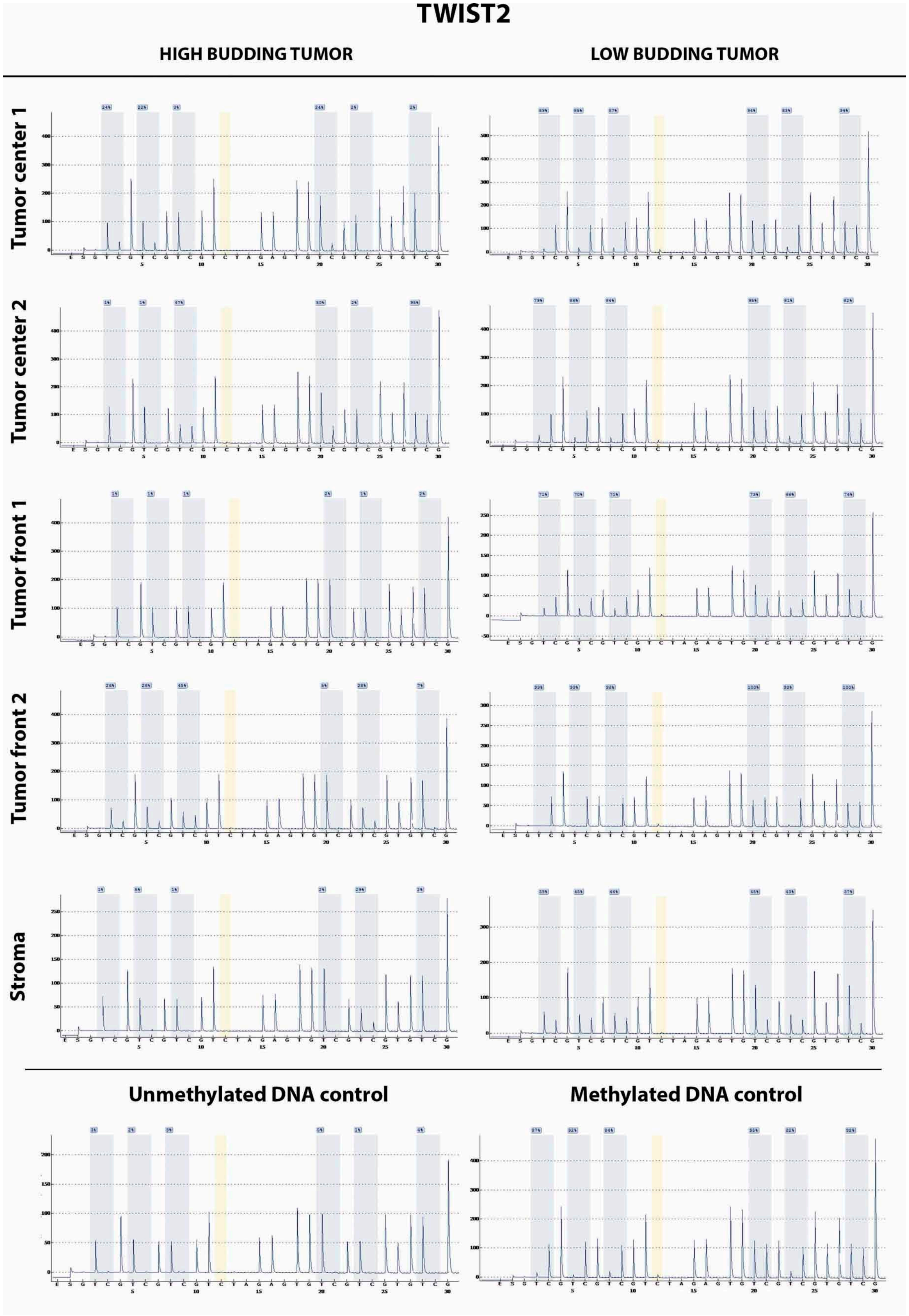

Supplementary Figure S2: Pyrograms showing successful TWIST2 methylation analysis of laser captured tumor center, tumor invasion front and tumor stroma in a low-grade and high-grade budding cancer

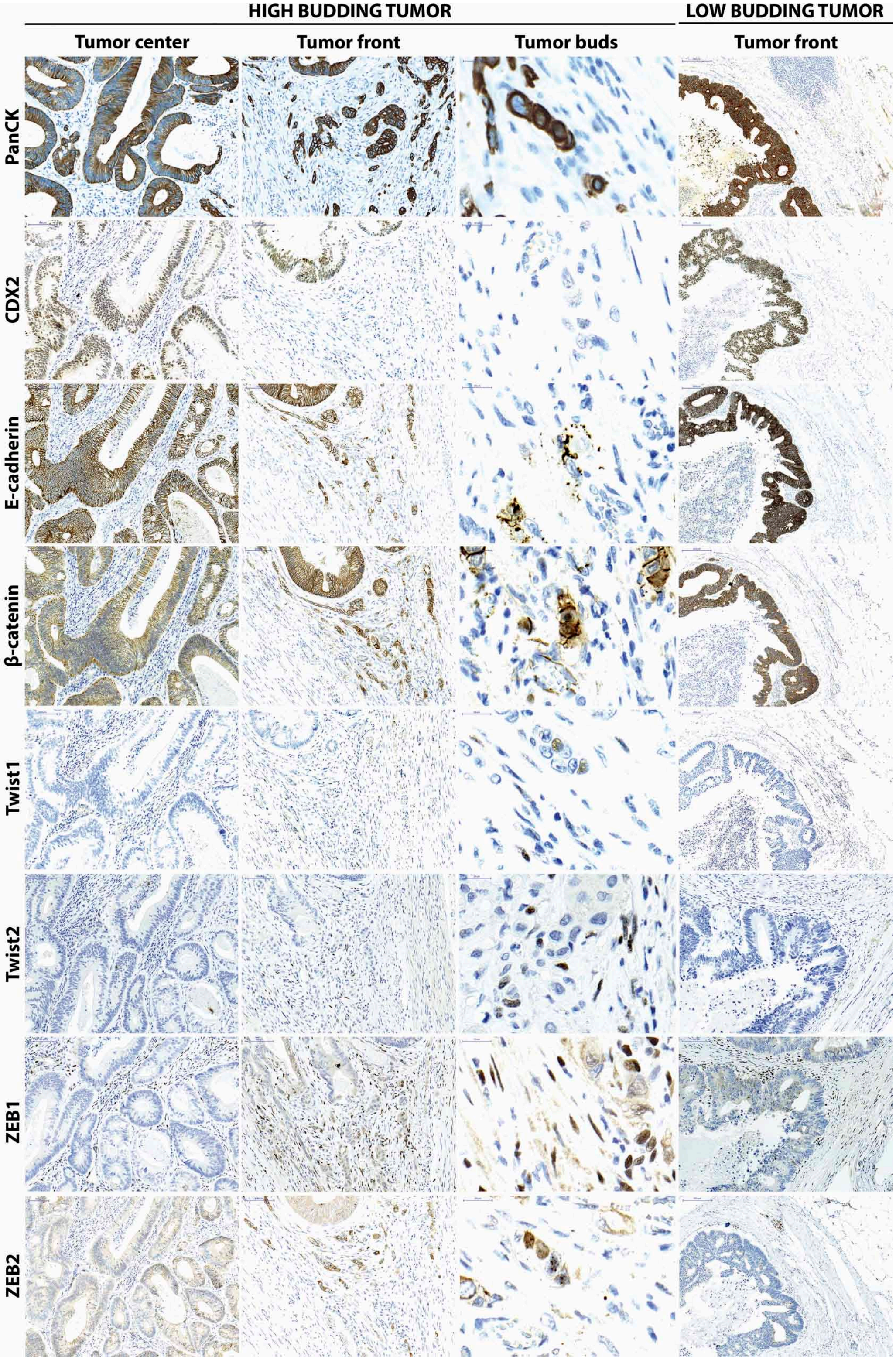

Supplementary Figure S3: Immunohistochemistry panel showing a high-grade budding cancer with focus on tumor center, invasion front (200x magnification, scale bar = 100 µm) and tumor buds (1000x magnification, scale bar = 20 µm), and a low-grade cancer (100x magnification, scale bar 200 µm). Staining includes β-catenin, E-cadherin, CDX2, TWIST1, TWIST2, ZEB1 and ZEB2.

Supplementary Table S1: Association of TWIST1 and TWIST2 hypermethylation and clinico-pathological features

Feature		Methylation TWIST1 (*n* = 109)		*P*-value	Methylation TWIST2 (*n* = 185)		*P*-value
		Low (*n* = 92)	High (*n* = 17)		Low (*n* = 80)	High (*n* = 105)	
Gender	Male	42 (45.7)	8 (47.1)	0.9148	40 (50.0)	49 (46.7)	0.653
	Female	50 (54.4)	9 (52.9)		40 (50.0)	56 (53.3)	
Histological subtype	Non-mucinous	81 (87.1)	16 (94.1)	0.4096	70 (86.4)	92 (87.6)	0.8088
	Mucinous	12 (12.9)	1 (5.9)		11 (13.6)	13 (12.4)	
Tumor grade	G1-2	58 (62.4)	12 (70.6)	0.517	48 (59.3)	70 (66.7)	0.2983
	G3	35 (37.6)	5 (29.4)		33 (40.7)	35 (33.3)	
pT	pT1-2	26 (28.0)	6 (35.3)	0.5402	16 (19.8)	31 (29.5)	0.1284
	pT3-4	67 (72.0)	11 (64.7)		65 (80.3)	74 (70.5)	
pN	pN0	47 (50.5)	6 (35.3)	0.2474	39 (48.2)	55 (52.4)	0.567
	pN1-2	46 (49.5)	11 (64.7)		42 (51.9)	50 (47.6)	
pM	pM0	80 (87.0)	15 (88.2)	0.8849	70 (86.4)	94 (90.4)	0.399
	pM1	12 (13.0)	2 (11.8)		11 (13.6)	10 (9.6)	
V	V0	73 (76.0)	15 (88.2)	0.2642	66 (80.5)	89 (83.2)	0.6333
	V1-2	23 (24.0)	2 (11.8)		16 (19.5)	18 (16.8)	
L	L0	52 (55.9)	9 (52.9)	0.8206	50 (61.7)	62 (59.1)	0.7111
	L1-2	41 (44.1)	8 (47.1)		31 (38.3)	43 (41.0)	
Budding	Low-grade	83 (88.3)	17 (100.0)	0.1373	39 (48.2)	65 (61.3)	0.0724
	High-grade	11 (11.7)	0 (0.0)		42 (51.9)	41 (38.7)	

Supplementary Table S2: Patient and tissue microarray characteristics

TMA characteristics		Cohort 1	Cohort 2
	Origin:	**Greece**	**Switzerland**
	Total no. patients	*N* = 215	*N* = 139
	TMA Punch size	0.6mm	1.0mm
	No. punches	4/tumor	1/tumor
	Patients included from:	2002–2006	2002–2011
	Surgical specimen/Biopsy	Surgery	Preoperative biopsy
**Clinicopathological features**			
Age (yrs)	Mean (min, max)	68.4 (35, 93)	70.2 (30–93)
Tumour size (cm)	Mean (min, max)	4.6 (1, 12)	-
Gender	Male	99 (48.3)	87 (63.0)

(*Continued*)

TMA characteristics		Cohort 1	Cohort 2
	Female	106 (51.7)	51 (37.0)
Histological subtype	Non-mucinous	181 (88.3)	116 (86.6)
	Mucinous	24 (11.7)	18 (13.4)
Tumour grade	G1-2	130 (63.4)	81 (68.1)
	G3	75 (36.6)	38 (31.9)
Tumour location	Left	124 (60.5)	43 (31.4)
	Rectum	27 (13.2)	39 (28.5)
	Right	54 (26.3)	55 (40.2)
pT	pT1+pT2	51 (24.9)	37 (26.8)
	pT3+pT4	154 (75.1)	101 (73.2)
pN0	pN0	103 (50.2)	69 (50.0)
	pN1-2	102 (49.8)	69 (50.0)
pM	pM0	183 (89.3)	98 (73.1)
	pM1	22 (10.7)	36 (26.9)
TNM stage	Stage I	41 (21.8)	24 (17.9)
	Stage II	53 (28.2)	34 (25.4)
	Stage III	76 (40.4)	40 (29.9)
	Stage IV	22 (9.6)	36 (26.9)
	TNM staging ed.	6th	7th
Tumour budding	Low-grade	115 (55.3)	71 (59.7)*
	High-grade	93 (45.7)	48 (40.3)
Venous invasion	Present	38 (18.1)	64 (53.3)
	Absent	172 (81.9)	56 (46.7)
Lymphatic invasion	Present	84 (40.6)	35 (29.9)
	Absent	123 (59.4)	82 (70.1)
Post-operative therapy	Untreated	76 (36.2)	108 (77.7)
	Treated	134 (63.8)	31 (22.3)
Pre-operative therapy	Untreated	210 (100%)	117 (86.0)
	Treated	0 (0%)	19 (14.0)
MMR status	Proficient	176 (91.7)	91 (85.1%)
	Deficient	16 (8.3)	16 (15%)
Survival time (months)	Median (95%CI)	60 (48-65)	101 (62-NE)

NE: Not evaluable

*In cohort 2, this value represents intratumoral budding (ITB), and not peritumoral budding
